# Evaluation of the ranawat triangle method for determining hip rotation center in total hip arthroplasty among Turkish patients

**DOI:** 10.1590/1806-9282.20260634

**Published:** 2026-10-23

**Authors:** Alper Dünki, Hakan Batuhan Kaya, Murat Birinci, Furkan Uçar, Ömer Polat, Savaş Çamur

**Affiliations:** 1 Umraniye Training and Research Hospital, Department of Orthopaedics and Traumatology – Istanbul, Turkey.

**Keywords:** Arthroplasty, Hip joint, Anatomic landmarks, Radiometry

## Abstract

**OBJECTIVE:**

Accurate identification of the anatomical femoral head center is critical in total hip arthroplasty for optimal implant positioning and long-term survival. The Ranawat triangle method is widely used for this purpose, but its applicability may vary across populations. The aim of this study was to evaluate the accuracy and suitability of the Ranawat triangle method in estimating the anatomical femoral head center in a Turkish population.

**METHODS:**

Standardized anteroposterior pelvic radiographs of 260 non-fractured hips from 312 patients aged 60–90 were retrospectively analyzed. Pelvic height, pelvic width, and femoral head diameter were measured. The coordinates of the anatomical femoral head center were calculated anatomically and using the Ranawat triangle method. Deviations between the two were assessed in both axes, with <5 mm considered acceptable error.

**RESULTS:**

Anatomical femoral head center estimated by the Ranawat triangle method was positioned an average of 4.7±1.5 mm medially and 1.0±0.8 mm cranially in men, and 4.8±1.1 mm medially and 1.1±0.7 mm cranially in women, relative to the anatomical center. While 100% of Y-axis errors and 58.1% of X-axis errors were within 5 mm. Strong positive correlations were found between pelvic height and Cy (r=0.59), and pelvic width and Cx (r=0.82).

**CONCLUSION:**

The Ranawat triangle method method demonstrated excellent accuracy in predicting the vertical (Y-axis) position of the anatomical femoral head center but showed moderate lateral (X-axis) deviations in over 40% of cases. Despite its limitations in lateral offset prediction, it may serve as a practical reference method for preoperative planning, particularly for estimating the vertical position of the hip rotation center in Turkish total hip arthroplasty patients.

## INTRODUCTION

Increasing life expectancy and the rising prevalence of osteoarthritis have led to a marked growth in the number of total hip arthroplasty procedures performed. Although this technique is highly successful, it is not entirely free of complications. Periprosthetic joint dislocations are the most common mechanical complications and are usually related to component placement^1,2^. Acetabular and femoral component position, combined anteversion, acetabular inclination, and femoral stem varus being appropriate for anatomic placement are important for long-term survival^3,4^.

The femoral head rotation center may not always be easily determined due to degeneration of the hip joint. For this reason, various methods have been determined and the Ranawat triangle method (RTM) is one of the most frequently used ones^5^.

Pelvic length, width, and anatomical femoral head center (AFHC) may vary according to race and gender^6^. Accordingly, there is no publication in the literature showing the applicability of the Turkish population with the RTM in determining AFHC. The aim of the study is to determine how accurately the RTM estimates in determining AFHC in Turkish patients.

## METHODS

Following Institutional Review Board (IRB) approval, pelvic radiographs of 260 patients were retrospectively reviewed. Radiological assessments were performed utilizing standardized anteroposterior (AP) pelvic radiographs. To establish a consistent reference system, orthogonal X- and Y-axes were defined at the outset. The X-axis was designated as the mediolateral line parallel to the plane connecting the most inferior margins of the bilateral teardrop figures, whereas the Y-axis was determined as the craniocaudal line drawn perpendicular to the X-axis, thereby forming a Cartesian coordinate system^6^.

In hips without radiographic evidence of arthrosis, the anatomical hip rotation center was identified through geometric fitting. Specifically, a best-fit circle was drawn to approximate the femoral head contour, and the circle’s center was designated as the anatomical rotation point. This center was subsequently used as the primary reference for all coordinate-based calculations, expressed as Cx and Cy along the X- and Y-axes, respectively^7^.

Pelvic width (PW) was measured on the X-axis as the distance between the lateral edges of the iliac crests. Pelvic height (PH) was defined on the Y-axis, extending from the highest point of the iliac crest to the lowest point of the ischial tuberosity. The vertical femoral head diameter (FHD) was also obtained on the Y-axis as the distance from the femoral head apex to its junction with the neck.

The AFHC was estimated using the method originally described by Ranawat et al.^8^. This technique approximates the true acetabular region by constructing an isosceles right triangle, in which both the base and height correspond to 20% of the measured PH. The triangle was positioned such that its inferior margin originated at the intersection of Kohler’s line and Shenton’s line. The AFHC was defined as the midpoint of the hypotenuse of this constructed triangle, and its coordinates were designated as Ax and Ay (Figure 1).

**Figure 1:**
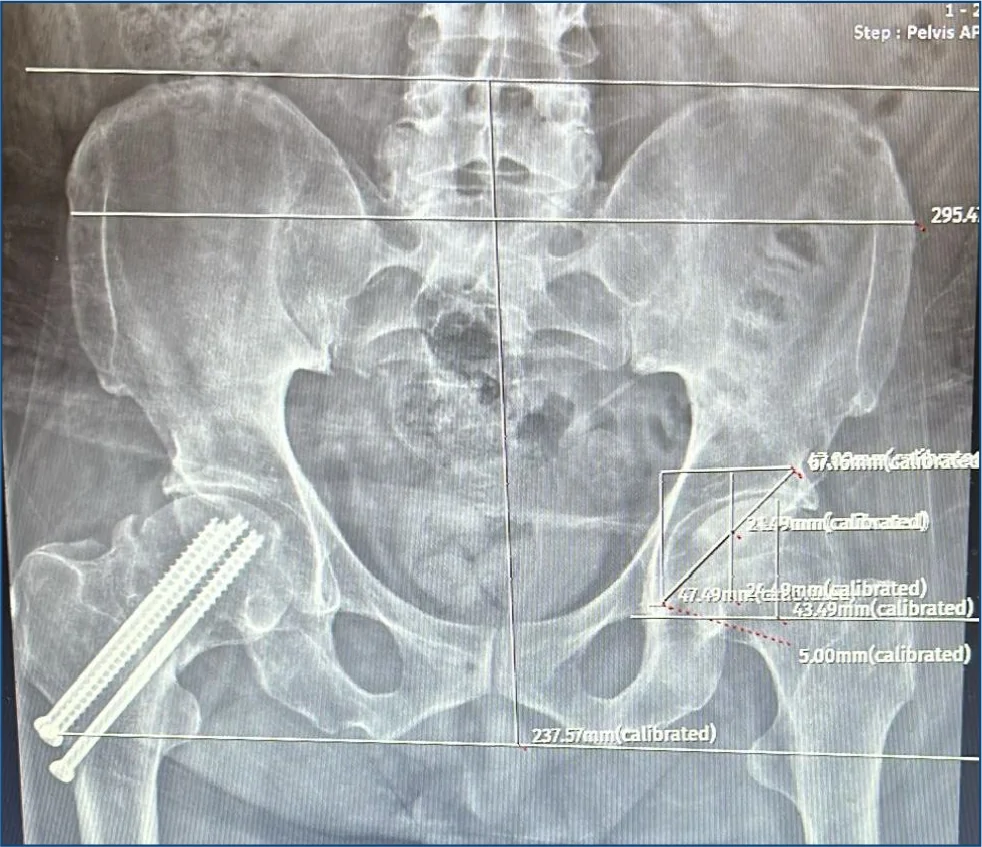
Measurement of the hip center of rotation on a calibrated anteroposterior pelvic radiograph using the Ranawat triangle method.

To evaluate anatomical alignment and method accuracy, several ratios and differences were calculated, including FHD/PH, Cx/PH, Cy/PH, Ax–Cx, and Ay–Cy. The RTM was considered reliable when the absolute differences between estimated and anatomical centers (Ax–Cx and Ay–Cy) were within a 5 mm threshold^6^.

All radiographic measurements were repeated twice by the first author with at least a 2-week interval to minimize intraobserver variation. Intraobserver reliability of all radiographic measurements was evaluated using the intraclass correlation coefficient (ICC). The analysis demonstrated an ICC value of 0.88, reflecting excellent reliability of the measurement technique. Data normality and variance homogeneity were tested with Shapiro-Wilk and F-tests. Student’s t-test or Wilcoxon rank-sum was applied for continuous variables, while categorical variables were assessed using the chi-square test. Associations were evaluated with Pearson correlation. Statistical significance was set at p<0.05, and analyses were performed with Statistical Package for the Social Sciences (SPSS) 29.0 (IBM Corp., Armonk, NY, USA).

Level of evidence: Level IV—Retrospective Observational Study.

## RESULTS

The mean Cx and Cy values were found to be 23.5±2.2 mm and 23.0±2.1 mm, respectively. Mean Cx, Cy, FHD, and PW were significantly greater in males compared to females, whereas no significant difference was observed in PH between sexes (Table 1). While the Cx/PH and Cy/PH ratios showed no significant gender-based differences, the FHD/PH ratio was higher in males than in females.

**Table 1:** Demographic and radiographic parameters.

	Men (n: 96)	Women (n: 164)	Statistical test	p
Age (y)	76.2±7.8	77.0±7.7	Mann-Whitney U	0.416
Laterality (L/R)	46/50	73/91	Chi-square test	0.595
LCEA (°)	46.1±9.2	45.6±10.5	Student’s t-test	0.698
Cx (mm)	24.3±2.4	23.1±2.0	Mann-Whitney U	<0.001
Cy (mm)	23.8±2.0	22.5±2.1	Student’s t-test	<0.001
PH (cm)	33.5±2.5	33.8±2.9	Student’s t-test	0.372
PW (cm)	24.0±1.9	22.9±1.6	Student’s t-test	<0.001
FHD (mm)	49.2±4.6	45.5±3.9	Student’s t-test	<0.001
Cx/PH	10.1±0.6	10.0±0.4	Mann-Whitney U	0.398
Cy/PH	9.9±0.5	9.8±0.5	Student’s t-test	0.318
FHD/PH	20.5±1.4	19.8±1.2	Mann-Whitney U	<0.001

cm: centimeter; FHD: femoral head diameter; mm: millimeter; n: sample size; PH: pelvic height; PW: pelvic width; y: year; °: angle; LCEA: lateral center edge angle.

The statistical evaluation indicated that PH showed a significant positive association with both coordinate values and femoral head morphology. Specifically, PH was positively related to the horizontal component (Cx, r=0.58, p<0.001), the vertical component (Cy, r=0.59, p<0.001), and the vertical FHD (r=0.58, p<0.001). PW also demonstrated strong associations with the same parameters. Higher PW values were linked to greater Cx (r=0.82, p<0.001), Cy (r=0.80, p<0.001), and FHD (r=0.72, p<0.001) measurements.

Application of the RTM yielded an estimated AFHC with mean coordinates of 28.3±1.8 mm along the Ax and 23.3±1.8 mm along the Ay. The mean deviation from the anatomical reference point was 4.7±1.3 mm in the mediolateral direction and 1.0±0.8 mm in the craniocaudal direction. These results demonstrate that the AFHC was consistently located medial and cranial to the true anatomical hip rotation center.

A subgroup analysis revealed sex-specific differences. In males, the AFHC was located approximately 4.7±1.5 mm medial and 1.0±0.8 mm cranial to the anatomical reference. In females, the displacement was similar, averaging 4.8±1.1 mm medially and 1.1±0.7 mm cranially relative to the true hip center (Table 1).

Regarding estimation performance, the mediolateral measurements exhibited substantial variability, with only 5.4% of all evaluated hips falling within a 3-mm range. In contrast, the craniocaudal assessments demonstrated markedly higher precision, as 98.8% of hips were within the same margin. When the threshold was extended to 5 mm, a distinct disparity between the two axes became apparent: 58.1% of hips fell within this limit along the X-axis, whereas all hips remained below the 5-mm deviation on the Y-axis (Table 2).

**Table 2:** Morphologic parameters.

	Men (n: 96)	Women (n: 164)	Statistical test	p
Ax (mm)	29.0±1.9	27.9±1.6	Student’s t-test	<0.001
Ax–Cx (mm)	4.7±1.5	4.8±1.1	Mann-Whitney U	0.401
|Error| <3 mm	8 hips	6 hips	Chi-square test	0.107
|Error| <5 mm	59 hips	92 hips	Chi-square test	0.398
Ay (mm)	24.0±1.9	22.9±1.6	Student’s t-test	<0.001
Ay–Cy (mm)	1.0±0.8	1.1±0.9	Mann-Whitney U	0.422
|Error| <3 mm	93 hips	160 hips	Chi-square test	0.742
|Error| <5 mm	96 hips	164 hips	–	–

mm: millimeter; n: sample size.

## DISCUSSION

Preoperative templating of the hip joint rotation center is important for long-term survival and reducing complications in hip arthroplasty. Various methods have been described to determine the correct hip rotation center in pelvic AP radiographs. However, due to morphological differences in population, not every method is suitable for every society^9^. In this study, the applicability of the RTM was examined in Turkish individuals with normal hip morphology. Measurements along the Y-axis remained within 5 mm in all patients. In contrast, 41.9% of hips demonstrated a displacement greater than 5 mm on the X-axis.

Takamatsu et al. found that the hip rotation center aligned with PH on the Y-axis and with PW on the X-axis^10^. In a similar study conducted with 100 THA patients in 2019, according to Yi et al., the position of the hip rotation center along the X- and Y-axes showed a positive association with PW and PH in Chinese individuals^11^. In a study conducted by Bombaci et al. in 2017 with 100 patients aged between 18 and 91, a positive correlation was observed between the distance of the location of the hip rotation center on the X-axis to the ischial tuberosity and the ratio of PW; the distance of the location on the Y-axis to the teardrop figure and PH in Turkish population^12^.

Fujii et al. reported that Cx showed a weak correlation with PW in the Japanese population in their study with 123 patients, but there was a strong correlation between Cy and PH. Fujii et al., who also determined the hip rotation center according to the RTM, found the estimation error in the X-axis as 6.7±2.6 mm and in the Y-axis as 6.6±1.9 mm. The accuracy of the estimations demonstrated that the absolute error values remained within a tolerance margin of 5 mm along both coordinate axes in a substantial proportion of cases, specifically in 118 hips (96%) for the X-axis and in 116 hips (94%) for the Y-axis, respectively^6^. The current study evaluates the hip rotation center in the Turkish population, and a strong positive correlation of Cx with PW and Cy with PH was observed. According to the RTM, 58.1% of the hips in the X-axis and Y-axis were below 5 mm in all patients, and the obtained data are consistent with the literature.

In examining the relationship between FHD and PH, Crowe et al. identified the FHD/PH ratio to be approximately 20%, forming the basis for their classification of hip dysplasia^13^. Similarly, Zhang et al, in a 2014 study involving 187 Chinese patients, determined the mean FHD/PH ratio to be 21.5% and concluded that the Chinese population also conforms to the Crowe classification^14^. In the present study, the FHD/PH ratio was measured as 20.5±1.4% in men and 19.8±1.2% in women, indicating that the Turkish population likewise aligns with the Crowe classification.

Various patient-specific methods have been described in the literature, including the RTM^8,15,16,17^. Schofer et al. evaluated the teardrop figure with the current method they applied to 115 patients in 2010. They stated that there was a non-negligible deviation with the RTM^18^. John et al. described in their study that the hip rotation center shifted medially and proximally with the RTM^19^. Fujii et al. also observed medial and proximal shifts in their study and described the modified RTM. In the current study, it was observed that the RTM had a negligible error in the Y-axis (mean 1.0±0.8 mm) and we observed that the hip rotation center shifted medially in the X-axis (mean 4.7±1.2 mm).

The study had some limitations. First, the study was conducted on patients who were candidates for THA in the Turkish population between the ages of 60 and 90. Second, the height, weight, and body mass index of the patients are unknown. Accordingly, PH, PW, and FHD values may vary. Finally, no matter how standardized the radiographs are, pelvic tilt and sacral slope have different values in each patient and AFHC coordinates, PH and PW values may vary in standard pelvic AP radiographs.

## CONCLUSION

The hip rotation center remained below 5 mm deviation in the Y-axis in all patients. Thus, the RTM showed considerable accuracy in predicting AFHC in THA cases. However, it showed poor accuracy in determining the lateral offset, with 58.1% deviation below 5 mm in the X-axis.

The RTM may be useful, albeit limited, in determining the hip rotation center in the placement of the acetabular component in THA cases applied to Turkish patients. Therefore, while the RTM can be reliably used to estimate the vertical position of the AFHC, surgeons should be aware of its tendency to underestimate the lateral offset and consider intraoperative or alternative radiographic adjustments when planning acetabular component placement. Given the frequent underestimation of lateral offset, precise preoperative template adjustments are essential to prevent postoperative gait dysfunction and joint instability. Clinicians should thus combine the RTM with anatomical landmarks to accurately restore proper lateral positioning.

## Data Availability

The datasets generated and/or analyzed during the current study are available from the corresponding author upon reasonable request.
